# Kidney fibrosis evaluation using dual-mode emission and transmission microscopy

**DOI:** 10.1117/1.JMI.13.6.067501

**Published:** 2026-11-05

**Authors:** Willy Ju, Samuel Border, Fatemeh Afsari, Srishti Seth, Sarah Rezapourdamanab, Ruben Renteria, Shalini Ramachandra, Rajib Gupta, Fadi Salem, Alton Farris, Richard Levenson, Jarcy Zee, Pinaki Sarder, Kuang-Yu Jen, Farzad Fereidouni

**Affiliations:** a Emory University, Department of Pathology and Laboratory Medicine, Atlanta, Georgia, United States; b University of Florida, Department of Biomedical Engineering, Gainesville, Florida, United States; c University of Pennsylvania Perelman School of Medicine, Department of Biostatistics, Epidemiology, and Informatics, Philadelphia, Pennsylvania, United States; d Mayo Clinic, Department of Pathology and Laboratory Medicine, Jacksonville, Florida, United States; e UC Davis Health, Department of Pathology and Laboratory Medicine, Sacramento, California, United States; f Children’s Hospital of Philadelphia Research Institute, Philadelphia, Pennsylvania, United States

**Keywords:** kidney, interstitial fibrosis, collagen, virtual staining, virtual trichrome, DUET microscopy, fluorescence, digital pathology, deep learning, transplant biopsy, interobserver agreement

## Abstract

**Purpose:**

Interstitial fibrosis is a critical histopathologic feature used in the diagnosis and prognosis of kidney diseases. However, conventional assessment using trichrome-stained slides is limited by interobserver variability and inconsistent stain reproducibility. This study evaluates whether dual-mode emission and transmission (DUET) microscopy, which generates virtual trichrome images from hematoxylin and eosin (H&E) slides, can provide a practical, reproducible, and quantitative alternative for assessing interstitial fibrosis in kidney transplant biopsies.

**Approach:**

Thirty-two kidney transplant biopsies were imaged using DUET to acquire pixel-registered brightfield and fluorescence whole-slide images from H&E-stained slides. A deep learning pipeline was trained on DUET-acquired images to extract collagen-positive signal from both the brightfield and fluorescence channels, which was used to generate virtual trichrome images and to compute quantitative fibrosis scores. Virtual trichrome images were compared with corresponding conventional trichrome slides. Four renal pathologists independently estimated interstitial fibrosis on both modalities to assess interobserver agreement, and computational fibrosis scores were correlated with pathologists’ estimates.

**Results:**

Pathologist assessments of fibrosis on DUET-derived virtual trichrome images demonstrated interobserver agreement comparable to traditional trichrome slides, trending higher for virtual trichrome (ICC 0.536 versus 0.477, p=0.376). Agreement of each pathologist with the other three readers was higher for virtual trichrome in three of the four pathologists and significantly higher in one. Fibrosis estimates were systematically lower on virtual trichrome than on real trichrome, by a mean of 7.6 percentage points. The deep learning model for collagen extraction achieved high segmentation performance (AUC 0.994, Dice 0.903). Computational fibrosis quantitation showed moderate correlation with pathologist estimates and demonstrated stronger association with virtual trichrome than real trichrome (r 0.67 versus 0.54, $R^{2}$ 0.44 versus 0.29).

**Conclusions:**

DUET microscopy enables the generation of pixel-registered virtual trichrome images from standard H&E slides with interobserver agreement comparable to conventional trichrome. By extracting collagen signal from pixel-registered fluorescence data on the same tissue section, DUET supports reproducible visual assessment and quantitative fibrosis measurement. This approach provides a scalable alternative to conventional trichrome staining and a foundation for integrated digital pathology workflows.

## 1 Introduction

Interstitial fibrosis (IF) is a common process of chronic kidney injury in all renal diseases.^1^ It is a key feature that renal pathologists routinely report to convey an estimated extent of irrecoverable renal injury, which has prognostic value for predicting the trajectory of future renal function. IF involves an excess accumulation of extracellular matrix that is primarily composed of collagen and, in essence, represents the extent of scar tissue within the kidneys. A large body of literature supports the strong association between the degree of IF and renal outcomes.^2^^–^^5^ As such, estimation of IF has been formally incorporated into guidelines for the classification of several renal diseases, including the Oxford classification for IgA nephropathy,^6^ the Banff classification for transplant rejection,^7^ and the International Society of Nephrology/Renal Pathology Society classification for lupus nephritis.^8^^,^^9^

Currently, it is standard practice for pathologists to use trichrome-stained slides to visually estimate the extent of IF. The trichrome stain helps to highlight collagen and is typically preferred over other histochemical stains. However, studies have shown that pathologists’ microscopic evaluation of IF is often hampered by problems with reproducibility.^10^^,^^11^ The trichrome stain is neither specific nor sensitive for collagen, leading to both underestimation of collagen content in cases of subtle or early fibrosis and overestimation of collagen content by nonspecifically staining interstitial edema or other structures.^10^^,^^12^^–^^14^ Technical variability of trichrome stains between laboratories as well as within the same laboratory can also affect the reproducibility of fibrosis estimation.^15^

Although conventional trichrome stains can be performed on permanent formalin-fixed paraffin-embedded (FFPE) biopsy sections, these special stains are generated from serial sections rather than the exact H&E slide used for primary diagnostic assessment. H&E sections provide critical morphologic context, including evaluation of inflammation, tubular injury, glomerular changes, and vascular pathology, whereas trichrome staining primarily highlights collagen deposition. Because serial sections are not identical at the microscopic level, the spatial distribution of collagen in a trichrome slide may not precisely correspond to the morphologic features observed on the adjacent H&E section. This lack of pixel-level correspondence can introduce challenges for computational models that rely on accurate alignment between fibrosis signals and underlying tissue architecture. Integrating collagen information directly on to the diagnostically informative H&E image, therefore, offers a potential advantage for both pathologist interpretation and computational analysis.

Recent efforts to create virtual trichrome stains using generative adversarial networks (GANs) have demonstrated the potential to reproduce trichrome-like appearances from standard H&E slides, with pathologist validation.^16^^,^^17^ However, these methods typically require large, high-quality training datasets and can be susceptible to artifacts or hallucinated structures inherent to generative modeling.

There is a clinical need for rapid and reliable visualization of collagen alongside histomorphological structures within a single slide to facilitate efficient and accurate pathological assessment of fibrosis. To address this need, we developed a novel microscopy approach termed Dual-mode Emission and Transmission (DUET). This whole-slide imaging technique scans standard H&E-stained slides in both brightfield and fluorescence modalities, enabling extraction of collagen signal from pixel-registered images within minutes. The resulting high-fidelity collagen signals can then be integrated with the H&E image to generate virtual trichrome images, eliminating the need for additional sections, stains, or labeling steps.^18^

We hypothesized that a meaningful component of interobserver variability in visual IF assessment is attributable to the trichrome stain itself, specifically its inconsistent reproducibility across laboratories and its reliance on subjective, color-based interpretation. Accordingly, we predicted that replacing conventional trichrome with a pixel-registered, computationally derived virtual trichrome image would support pathologist agreement at least as well as conventional trichrome. We hypothesized specifically that inter-pathologist agreement, quantified by the intraclass correlation coefficient (ICC), would be comparable to or better than that obtained with conventional trichrome.

In this study, we apply DUET to transplant kidney biopsies, producing a pixel-registered collagen image and virtual trichrome whole-slide images that renal pathologists assessed to demonstrate the practical utility of this imaging technique. Furthermore, we developed a semi-automated computational pipeline for estimating IF in these kidney biopsies using the collagen image extracted from DUET data. Our approach aims to address the challenges of traditional clinical and computational fibrosis estimation by deploying a whole slide-capable, accurate, quantitative, and reproducible method for assessing IF in kidneys, leveraging complementary information from both brightfield and fluorescence imaging modalities.

## 2 Methods

### 2.1 DUET Image Acquisition

Pixel-registered brightfield and fluorescence whole-slide images were acquired from FFPE H&E stained slides using the DUET imaging system. For fluorescence imaging, epifluorescent excitation was employed with a 405 nm LED light source (LED Engin, LZ1-00UB00, San Jose, California, United States), directed toward the specimen through a broadband dichroic mirror (Semrock, Di03-R405-t1-25×36) and focused using a 20× objective lens (Nikon, NA=0.75). Fluorescence emission was collected back through the same dichroic mirror, with residual excitation light rejected by a long-pass emission filter (Semrock, FF01-430/LP-25). The emission filter sets the short-wavelength limit of the detection band, and the sensor response sets the long-wavelength limit, so emission was recorded from approximately 430 to 700 nm. The emitted fluorescence signal was captured with a nine-megapixel scientific-grade CCD color camera (Ximea, MD091CU-SY) coupled to a 200 mm tube lens (Thorlabs, ILT 200, Newton, New Jersey, United States). The detector is a single-chip color CCD with a Bayer filter array, so each pixel records one of three color bands. Over the detection band, the approximate spectral response of the three channels is red 600 to 700 nm, green 450 to 600 nm, and blue 430 to 500 nm. These three channels constitute the spectral data used for the phasor analysis described in Fig. 1. Both brightfield and fluorescence channels were acquired using an automated XYZ motorized stage (Zaber, X-ASR050B050B-SD12 and X-DMQ12L-AE55D12), controlled by custom software written in VB.NET.

**Fig. 1:**
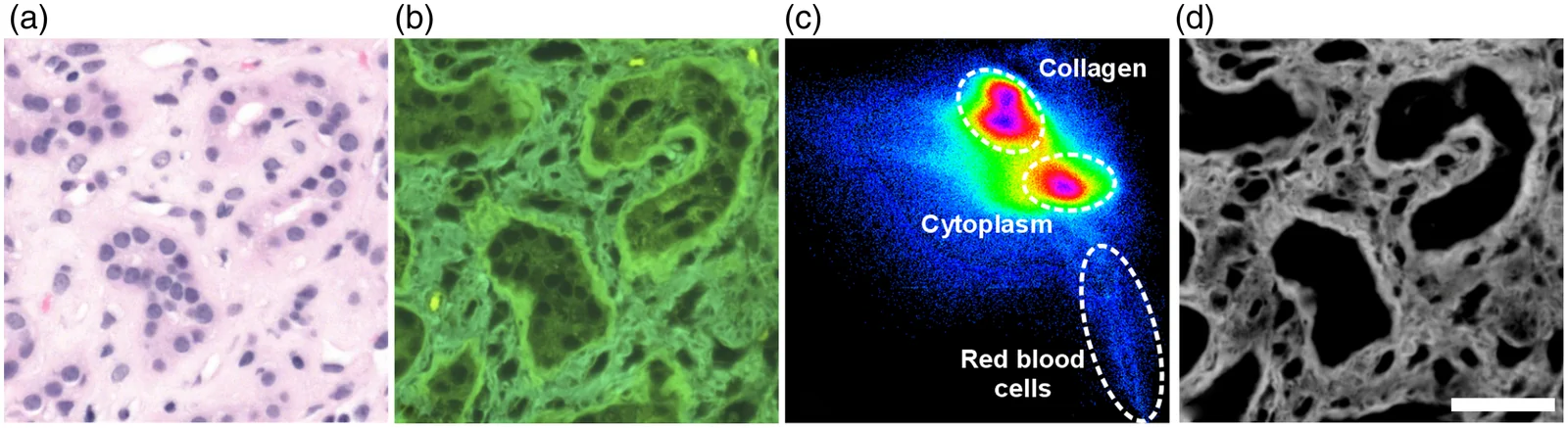
Generation of collagen ground-truth labels using a spectral phasor approach. (a) Representative DUET brightfield image of an H&E-stained kidney section. (b) Representative DUET fluorescence image of an H&E-stained kidney section. (c) Spectral phasor plot showing distinct clusters corresponding to collagen, cytoplasm, and red blood cells used to identify collagen-positive pixels in the fluorescence image. (d) A collagen mask derived from the spectral phasor analysis was subsequently used as ground truth for training the automated collagen extraction. Scale bar=50  μm (applies to all images).

### 2.2 Case Selection

Thirty-two core needle transplant kidney biopsy cases were selected from the UC Davis Health clinical archive by a renal pathologist (K.-Y.J.). Cases were chosen to span the full range of interstitial fibrosis, from minimal to advanced, and included cases with inflammatory infiltrates. Because transplant biopsies with greater than 50% fibrosis are uncommon in routine practice, such cases were deliberately selected to cover the upper end of the range. Cases were also required to have an H&E section and a serial trichrome section from the same block, both of adequate staining quality, and sufficient cortex for fibrosis assessment. The biopsies were performed between 2016 and 2020. Selection was carried out on the original glass slides before any virtual trichrome image was generated. Of the 32 cases, 23 were sourced from the original H&E and trichrome slides used for routine clinical care, sectioned and stained by the UC Davis Health Pathology Laboratory. The remaining nine cases were generated from the original tissue blocks but stained in a different research laboratory (UC Davis Center for Genomic Pathology Lab). For one case, we included a visual comparison of the H&E, real trichrome, extracted collagen, and virtual trichrome images from the two different laboratories, all originating from the same formalin-fixed paraffin-embedded tissue block.

Transplant biopsies were used for two reasons. Interstitial fibrosis scoring is standardized in transplant pathology through the Banff classification, which provides a widely adopted grading framework against which a new modality can be evaluated. In addition, native kidney disease spans a broad range of entities, each with its own classification scheme and characteristic distribution of chronic injury, and fibrosis is often skewed within a given entity, so assembling a native cohort covering the full fibrosis range would require a substantially larger study than the one reported here.

This study was conducted in accordance with the principles of the Declaration of Helsinki and was approved by the Institutional Review Board (IRB) at the University of California, Davis (Protocol #2013364). The study utilized archived, de-identified human kidney transplant biopsy specimens obtained from the clinical pathology archives of UC Davis Health. The use of archived, de-identified specimens qualified for a waiver of informed consent as determined by the IRB, as the study posed minimal risk and involved no direct patient contact. All research procedures were conducted in accordance with relevant institutional and regulatory guidelines.

### 2.3 Automated Collagen Image Extraction Using Deep Learning

The fluorescence image contains clear signals corresponding to basement membranes and bulk collagen surrounding glomeruli and renal tubules, features composed mainly of collagen types III and IV (Fig. 1). These basement membrane structures are critical for evaluating tubular atrophy and interstitial expansion, both of which are central to fibrosis scoring. However, such features are often difficult to resolve using standard brightfield imaging alone. By capturing these signals in the fluorescence channel, DUET enables more precise localization of collagen within the tissue architecture, especially in areas where fibrosis may be subtle or early. Although DUET fluorescence signals are inherently continuous, binary collagen masks were generated for segmentation evaluation and computational quantitation.

To automate the collagen extraction process, ground truth labels were generated using our previously published spectral phasor approach,^18^^–^^20^ as shown in Fig. 1. Spectral phasor analysis is based on the Fourier transformation of spectral data.^19^ Here the spectral data are the three color channels recorded by the camera. For each pixel, the discrete Fourier transform of the three channels is calculated, and the real (G) and imaginary (S) components of the first harmonic are plotted on a two-dimensional phasor plot. Pixels with similar spectral signatures occupy similar positions in phasor space, so tissue components with different emission color form distinct clusters. Collagen, cytoplasm, and red blood cells formed separable clusters (Fig. 1(c)), and the pixels in the collagen cluster were mapped back to the image to produce the collagen mask (Fig. 1(d)). This allows segmentation of tissue components by spectral property without pixel-by-pixel classification. The spectral phasor approach allows signals from collagen and cytoplasm to be separated based on the color difference between collagen and cytoplasm in the fluorescence images. The segmentation was performed automatically by identifying the individual clusters and performing a reciprocal transformation to find the pixels associated with each cluster. The cluster assignments were reviewed visually by a member of the study team (F.F.), and minor manual adjustments to the cluster boundaries were made to improve separation between components. No model was trained at this step. The resulting collagen labels were reviewed under renal pathologist guidance (K.-Y.J.) and verified against second-harmonic generation (SHG) imaging on one slide.

Segmentation performance was evaluated against the phasor-derived collagen masks described above, which served as the reference standard for both training and evaluation. The metrics in Table 1 were calculated on the held-out 20% of patches by comparing the binarized model output with the corresponding reference mask pixel by pixel.

**Table 1:** Performance metrics for collagen segmentation using concatenated brightfield and fluorescence inputs.

Metric categories	Segmentation model performance (mean ± 1 SD)
AUC	0.994±0.05
Sensitivity	0.899±0.09
Specificity	0.875±0.15
Dice	0.903±0.06
Accuracy	0.908±0.06

Performance metrics for collagen segmentation are shown, including area under the receiver operating characteristic curve (AUC), sensitivity, specificity, Dice coefficient, and accuracy. All metrics were calculated on held-out image patches by comparing the binarized model output with the reference collagen mask generated by the supervised spectral phasor approach, which served as the ground truth for training and evaluation.

No independent absolute standard for interstitial collagen was available, so these metrics reflect agreement between the model and this reference. The two machine learning models used in this study, the data on which each was trained, and the source of the corresponding ground truth labels are summarized in Supplemental Table S1.

Seven hundred image patches from three cases with a different range of fibrosis were used, and the output of clustering brightfield and fluorescence images was used for training a UNet++ model. The input dimensions for training were 512×512  pixels. A UNet++ model architecture was employed, utilizing the Adam optimizer with an initial learning rate of 5e-5 and 1000 training steps, with a batch size of 2. The training images comprised 80% of the original annotated image dataset.

### 2.4 Virtual Trichrome Creation

The green channel was extracted from each of the pixel-registered brightfield and fluorescence images and used as the two input channels to the UNet++ model, which produced the collagen image. Only the green channel was used, which reduces the effect of color variation between slides, staining batches, and laboratories on the model input. Because the objective at this step is to localize collagen rather than to distinguish hematoxylin from eosin, a single intensity channel from each modality provides a more consistent input than the full RGB image. The two channels carry complementary information: collagen is strongly fluorescent, while hematoxylin is essentially nonfluorescent. The fluorescence channel provides contrast between collagen and the surrounding tissue, and the brightfield channel provides the tissue morphology in which that signal is located. The spectral phasor analysis described above was used only to generate the training labels; once trained, the model operates on the two green-channel images without any phasor step. Next, the collagen image was pseudo-colored and overlaid onto a synthetic trichrome base derived from the brightfield image. Figure 2 illustrates this process, showing the brightfield and fluorescence inputs, the intermediate collagen and trichrome base images, the final virtual trichrome, and a serially-sectioned real trichrome image for comparison. Because the virtual trichrome is generated from the same section as the H&E image, the two are inherently registered, and the virtual trichrome can be produced in whole-slide format for direct comparison with the original H&E layer.

**Fig. 2:**
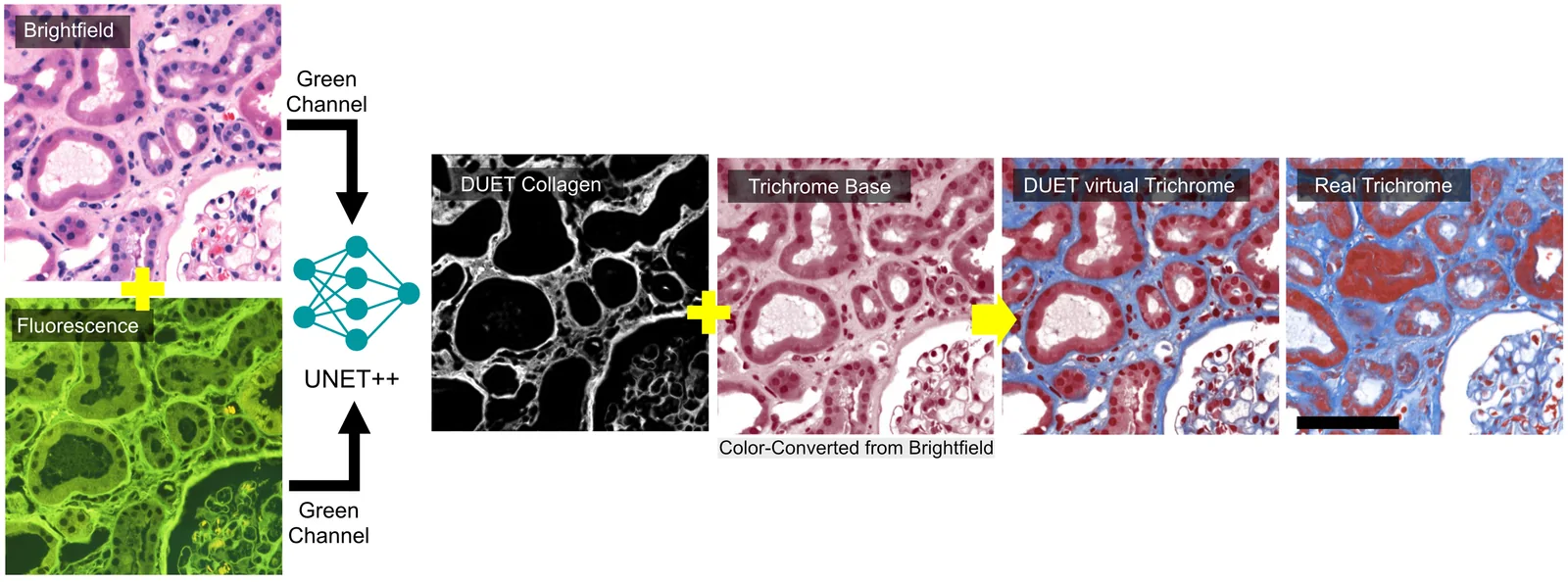
Workflow for generating virtual trichrome images from DUET imaging. Virtual trichrome images were generated using pixel-registered brightfield and fluorescence images acquired from the same H&E-stained slide as inputs. The green channel was extracted from each image and input into a UNet++ deep learning model to generate a collagen image. A trichrome base image was synthesized from the brightfield channel, and the DUET-derived collagen image was overlaid onto this base to create the virtual trichrome. For comparison, a real trichrome image from a serial section of the same tissue region was acquired using a conventional brightfield scan. Scale bar=100  μm (applies to all images).

### 2.5 Pathologist Fibrosis Estimation

Four renal pathologists at three academic institutions, with 8 to 22 years of renal pathology practice experience, estimated the percentage of renal cortical IF involvement to the closest 5% on 32 real trichrome whole slide images (set 1) and their corresponding virtual trichrome whole slide images (set 2). The order of the cases for each set was randomized, and a 2-week washout period was implemented between evaluation of the sets. The virtual trichrome images were generated from the H&E slides in closest proximity to the real trichrome slides in terms of depth within the paraffin block.

### 2.6 Statistical Analysis

Because each sample was rated twice per pathologist, once for real trichrome and once for virtual trichrome, the intraclass correlation coefficient (ICC) was calculated within subgroups. The ICC was calculated as a ratio of between-sample variability to the sum of between- and within-sample variability, based on a random effects model, and is therefore bounded by zero. About 95% parametrically bootstrapped confidence intervals were calculated and reported for each ICC using the bootMer function in the lme4 R package with 1000 iterations.

To test whether the ICCs between Trichrome and vTrichrome were statistically significantly different, clustered bootstrapping was used by resampling samples with replacement with 1000 iterations. The two-sided p-value was calculated as the proportion of bootstrap deviations at least as extreme as the observed difference. Differences were considered statistically significant at p<0.05.

Agreement between the two modalities was additionally examined by Bland–Altman analysis. For each pathologist and case, the difference between the real and virtual trichrome score (real minus virtual) was plotted against the mean of the two scores. The mean difference was taken as the systematic offset between modalities, and 95% limits of agreement were calculated as the mean difference ± 1.96 standard deviations of the differences. Because each case was read by all four pathologists, these limits describe the spread of individual paired readings rather than of independent observations, so the offset was tested by a paired t test on the case-level mean differences (n=32), and a case-level version of the plot is provided in Supplemental Fig. S1. Dependence of the difference on fibrosis severity was assessed by regressing the difference on the mean of the two scores.

The relationship between the computational fibrosis score and the average pathologist estimate was summarized by the Pearson correlation coefficient (r), with the coefficient of determination ($R^{2}=r^{2}$) reported alongside it. These quantities describe the strength of association between the two measurements and were not interpreted as measures of agreement, because the computational score and the pathologist estimate are not on a common scale.

### 2.7 Multiclass Segmentation of Glomeruli and Arteries

To isolate the interstitial compartment for fibrosis quantification, a multiclass segmentation model was trained to identify glomeruli and large arterial structures. The model was based on a U-Net architecture and accepted two-channel inputs consisting of the green channel from pixel-registered brightfield and fluorescence images. This segmentation is used only to exclude glomeruli and large arteries from the computational fibrosis score and does not affect the virtual trichrome images.

Training data were derived from four cases (WSIs), which were divided into 4096×4096 tiles and downsampled to an effective input size of 1024×1024  pixels (resize scale=0.25). Manual annotations of glomeruli and arteries were generated from paired brightfield and fluorescence image stacks using FIJI/ImageJ and converted into multiclass segmentation masks. A total of 363 annotated patches were used, with 252 patches for training and 111 for validation using a stratified 70/30 split. The model was trained using a combination of weighted multiclass cross-entropy loss and multiclass Dice loss, with the Dice component applied only to foreground classes. Optimization was performed using AdamW with an initial learning rate of 0.001. The model was trained for 15 epochs, and the model was selected based on the validation foreground Dice.

Predicted glomeruli and artery regions were excluded from downstream fibrosis quantification. Postprocessing was applied to remove small spurious predictions with a minimum blob size threshold (min blob area=250,000  pixels), while applying a Gaussian blur (σ=25.0) to smoothen prediction masks after thresholding.

### 2.8 Computational Interstitial Fibrosis Quantitation

Computational interstitial fibrosis was quantified as the proportion of DUET-extracted collagen-positive pixels relative to the total cortical area. Glomeruli and large arteries were excluded using a multiclass segmentation model (Fig. 3(c)), while noncortical regions were excluded by manual annotation performed by a renal pathologist (K.-Y.J.). The resulting computational IF measurements were then compared with pathologists’ visual estimates of fibrosis.

**Fig. 3:**
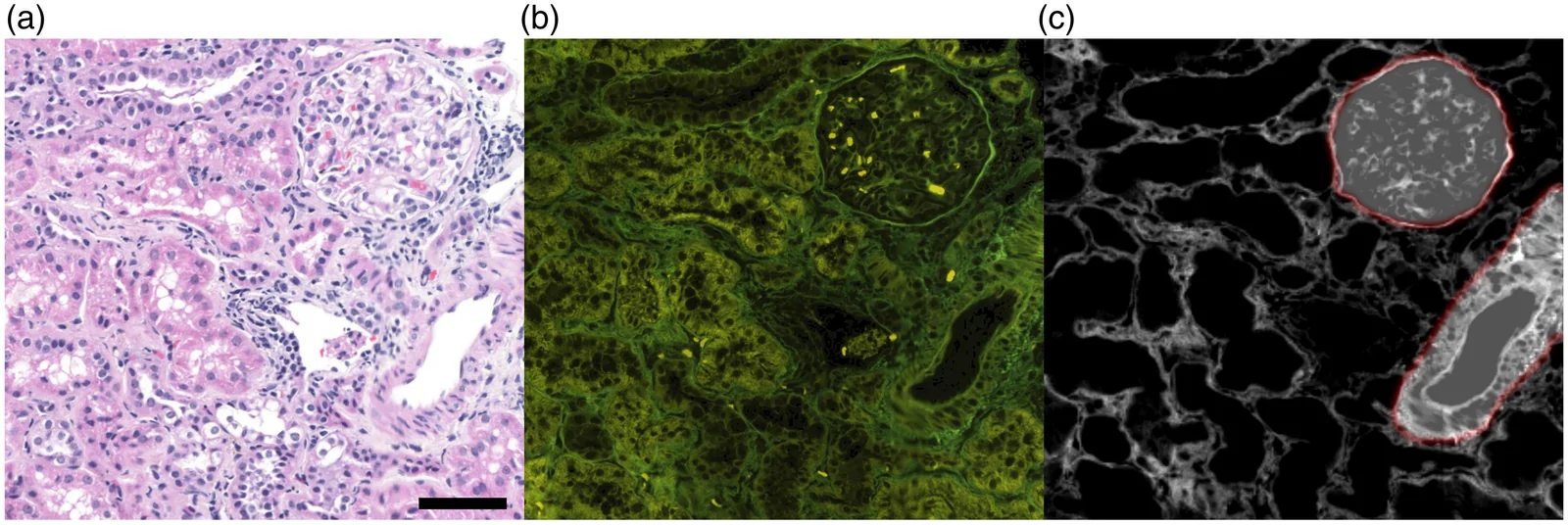
Computational interstitial fibrosis scoring excluding glomeruli, large arteries, and non-cortex areas. Representative H&E-stained kidney biopsy showing inputs and outputs for the computational fibrosis approach, with glomeruli and large arteries excluded. (a) Representative brightfield image of an H&E-stained kidney section. (b) Representative DUET fluorescence image of an H&E-stained kidney section. (c) Extracted collagen image showing excluded glomeruli and larger artery areas in red outline. Scale bar=50  μm (applies to all images).

## 3 Results

### 3.1 Performance of Automated Collagen Signal Processing from DUET Images

Collagen prediction images were generated as 2D images, which were then binarized before calculating segmentation metrics. The model achieved AUC (0.994±0.05), sensitivity (0.899±0.09), specificity (0.875±0.15), Dice (0.903±0.06), and accuracy (0.908±0.06) (Table 1).

Performance metrics for collagen segmentation are shown, including area under the receiver operating characteristic curve (AUC), sensitivity, specificity, Dice coefficient, and accuracy. All metrics were calculated on held-out image patches by comparing the binarized model output with the reference collagen mask generated by the supervised spectral phasor approach, which served as the ground truth for training and evaluation.

Examples of predicted collagen images from various kidney features, including tubules, interstitium, glomeruli, inflammation, and arteries, are compared with the corresponding brightfield and fluorescence images in Fig. 4.

**Fig. 4:**
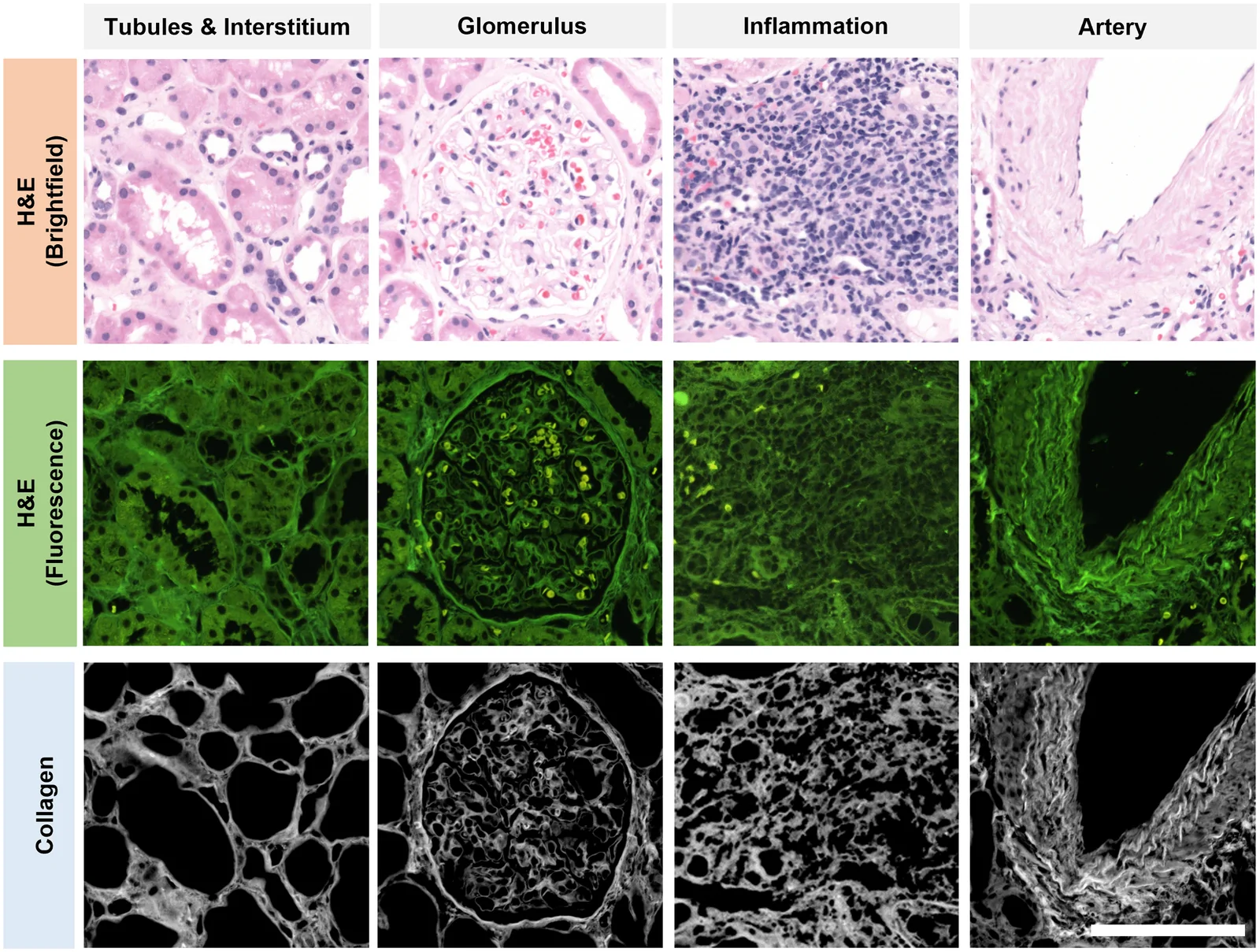
Example image patches of brightfield and fluorescence inputs with predicted collagen masks across kidney tissue features. Example 512×512 image patches of brightfield and fluorescence inputs shown with predicted collagen masks of different kidney features, including: tubules and interstitium, glomerulus, inflammation, and arteries. Scale bar=100  μm (applies to all images).

### 3.2 Virtual Trichrome Evaluation

From the DUET-derived collagen signals, virtual trichrome images were generated. To assess robustness under variable staining conditions, H&E- and trichrome-stained serial sections from the same kidney specimen were compared between two laboratories [Figs. 5(a)–5(h)]. Qualitatively, the H&E images from the two laboratories differ noticeably in color profile and intensity [Figs. 5(a) and 5(e)]. The corresponding real trichrome stains also vary in appearance: Lab A’s trichrome shows stronger contrast between fuchsinophilic (red-staining) tubules and blue-staining collagen [Fig. 5(b)], whereas Lab B’s trichrome provides clearer visualization of basement membranes and capillary structures within the glomerulus [Fig. 5(f)]. Yellow arrows in Fig. 5 indicate the tubular basement membrane, and green arrows indicate the basement membrane surrounding the glomerulus (Bowman’s capsule).

**Fig. 5:**
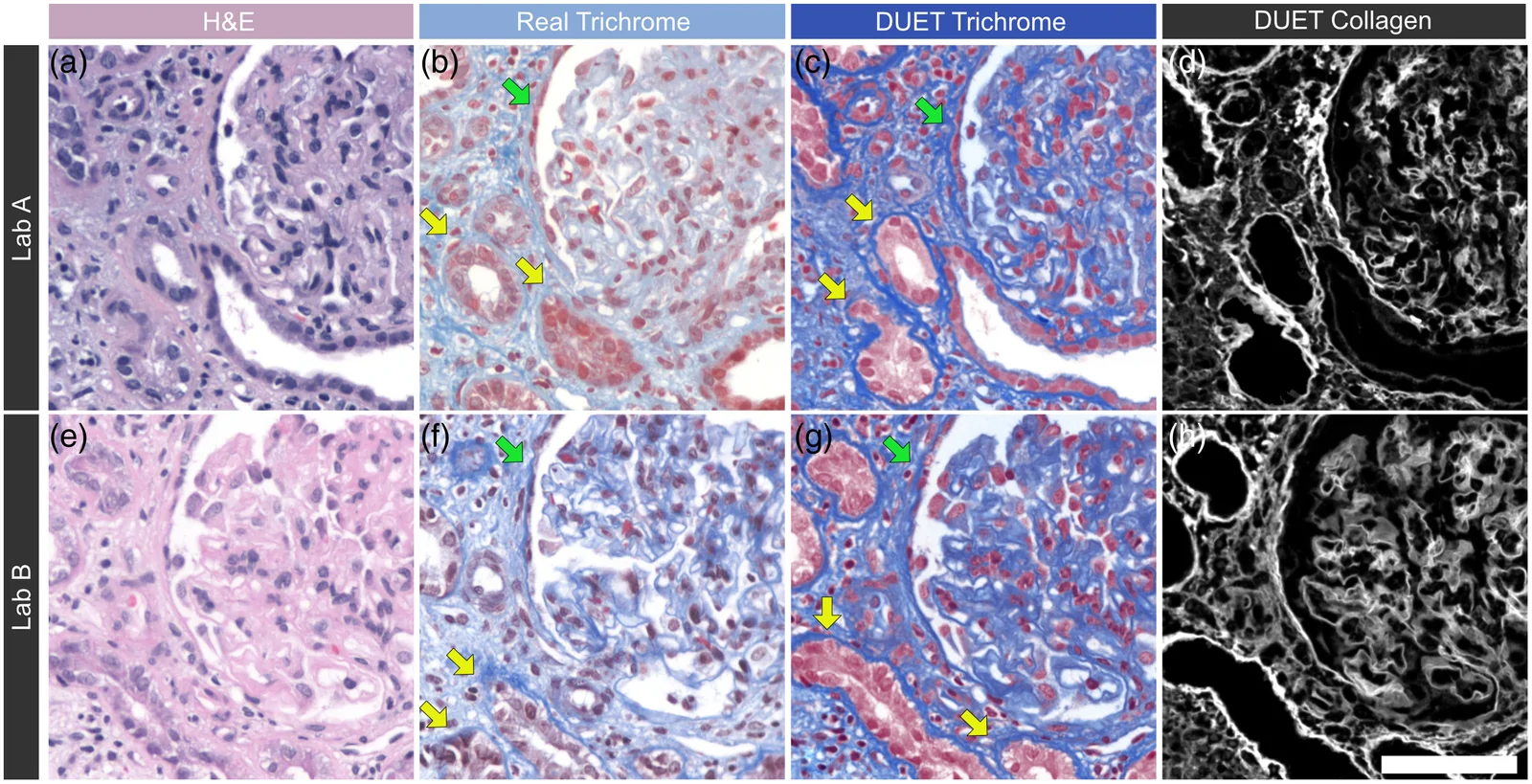
Comparison of conventional trichrome staining and DUET-derived virtual trichrome across H&E slides from two laboratories. Representative kidney H&E (a,e), real trichrome (b,f), DUET virtual trichrome (c,g), and DUET-extracted collagen (d,h) images from two different labs (Lab A, row 1; Lab B, row 2) at UC Davis. Yellow arrows indicate the tubular basement membrane, and green arrows indicate the basement membrane surrounding the glomerulus (Bowman’s capsule), shown in the real and virtual trichrome panels (b,c,f,g). The Lab A trichrome (b) shows stronger contrast (red tubules versus turquoise collagen), whereas the Lab B trichrome (f) shows better spatial resolution and specific binding; for example, the glomerular basement membrane is more distinctly visualized (f). In contrast, the DUET trichrome (c,g) provides a consistent color theme and quality across both H&E sources. Collagen distributions extracted from the H&E images (d,h) remain of similar quality despite differences in staining. Scale bar=100  μm (applies to all images).

In contrast, the DUET-generated virtual trichrome images remain visually consistent across both sources [Figs. 5(c) and 5(g)]. Despite variation in the original H&E stains, the virtual trichrome images were judged on a qualitative side-by-side review to retain comparable color appearance and structural detail, particularly along glomerular and tubular basement membranes. No quantitative metric of color contrast was computed; this assessment was made by the renal pathologists among the study authors on side-by-side review of the paired whole-slide images. The collagen distributions extracted from DUET fluorescence images likewise maintain comparable structural quality across both laboratories [Figs. 5(d), and 5(h)], appearing largely unaffected by differences in the initial H&E staining.

Figure 6 compares real (a, c) and DUET-derived (b, d) trichrome stains. The fields of view are 0.89×0.89  mm (a, b) and 0.22×0.22  mm (c, d). The virtual images demonstrate qualitatively improved color contrast, structural detail, and visualization of collagen; arrows mark the tubular and glomerular basement membranes in both modalities.

**Fig. 6:**
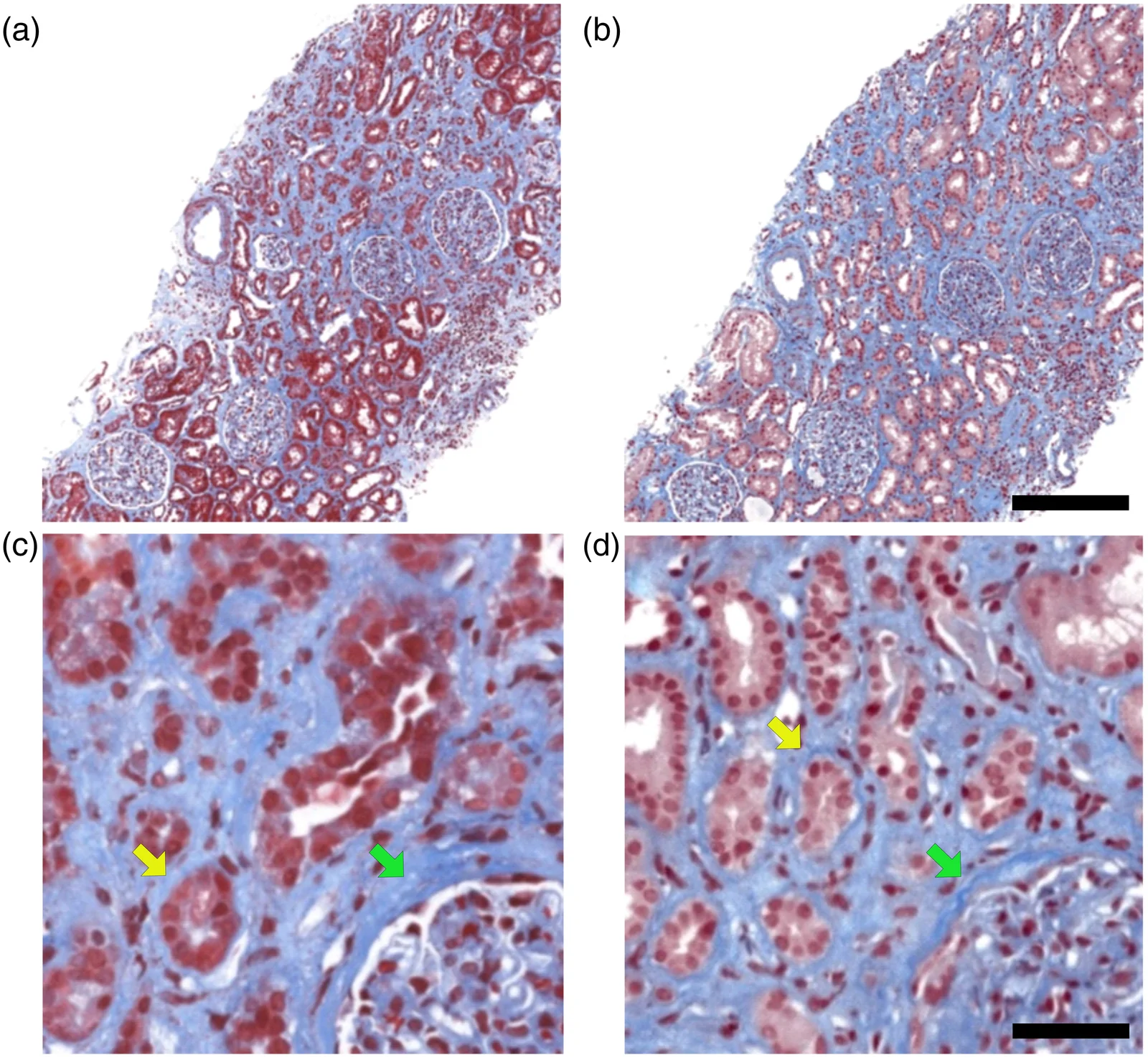
Low- and high-power comparison of real trichrome and DUET-derived virtual trichrome from the same kidney biopsy region. Panels (a) and (c) show traditional trichrome stains highlighting interstitial collagen in blue and cytoplasm in red. Panels (b) and (d) show the corresponding virtual trichrome images generated from DUET, replicating the color contrast, structural detail, and visualization of the real trichrome. Yellow arrows indicate the tubular basement membrane and green arrows the glomerular basement membrane, shown in both the real and virtual trichrome panels. All images were acquired with a 20× objective (NA 0.75). Panels (a) and (b) span a field of approximately 0.89×0.89  mm; panels (c) and (d) span approximately 0.22×0.22  mm, corresponding to approximately 95× and 380× at a printed panel width of about 84 mm. (a)-(b) Scale bar=200  μm. (c)-(d) Scale bar=50  μm.

### 3.3 Pathologist Interstitial Fibrosis Estimation of Real and Virtual Trichrome Stains

To assess the utility of DUET-derived virtual trichrome stains, four renal pathologists visually estimated the extent of IF in a set of 32 real trichrome whole slide images and their corresponding virtual trichrome whole slide images. Paired scores from the two modalities were positively correlated for every pathologist (Fig. 7; per-pathologist r=0.69 to 0.80, pooled r=0.80), with more points falling below the line of perfect agreement, reflecting slightly higher estimates on real trichrome.

**Fig. 7:**
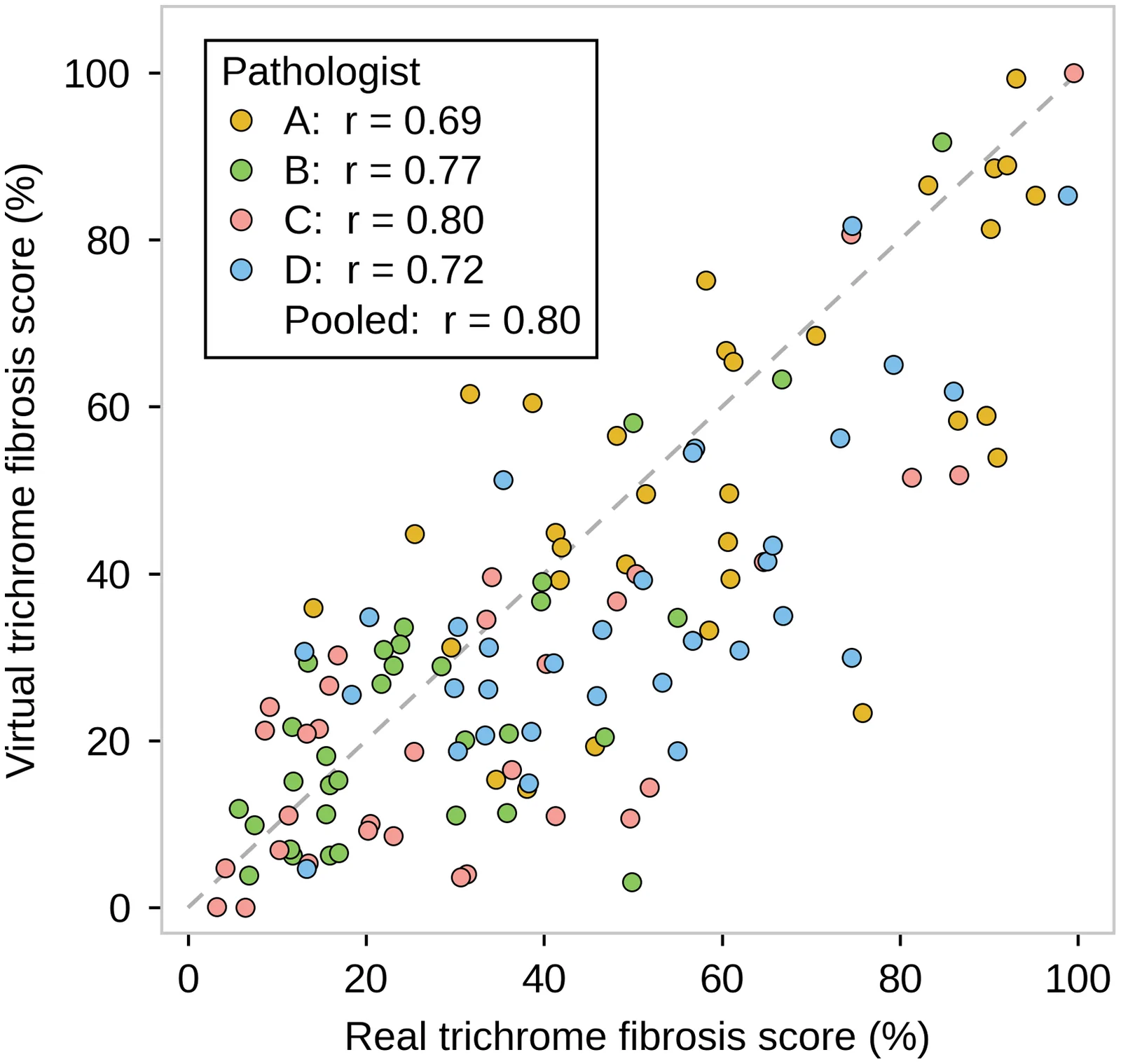
Agreement between virtual and real Masson’s trichrome fibrosis scores. Each point is one pathologist’s score of one kidney biopsy case (32 cases each scored by four pathologists, 128 paired observations); virtual trichrome fibrosis score (%) is plotted against the corresponding real trichrome score (%), with points color-coded by pathologist (A–D). The dashed line indicates perfect agreement (y=x). Pearson correlation coefficients are shown for each pathologist individually and for all 128 paired observations pooled (r=0.80).

Case-level mean scores followed the same ordering in both modalities across the full fibrosis range (Fig. 8). Spread among the four readers was comparable in the two modalities, with a mean across-reader standard deviation of 17.2 percentage points for real trichrome and 15.5 for virtual trichrome.

**Fig. 8:**
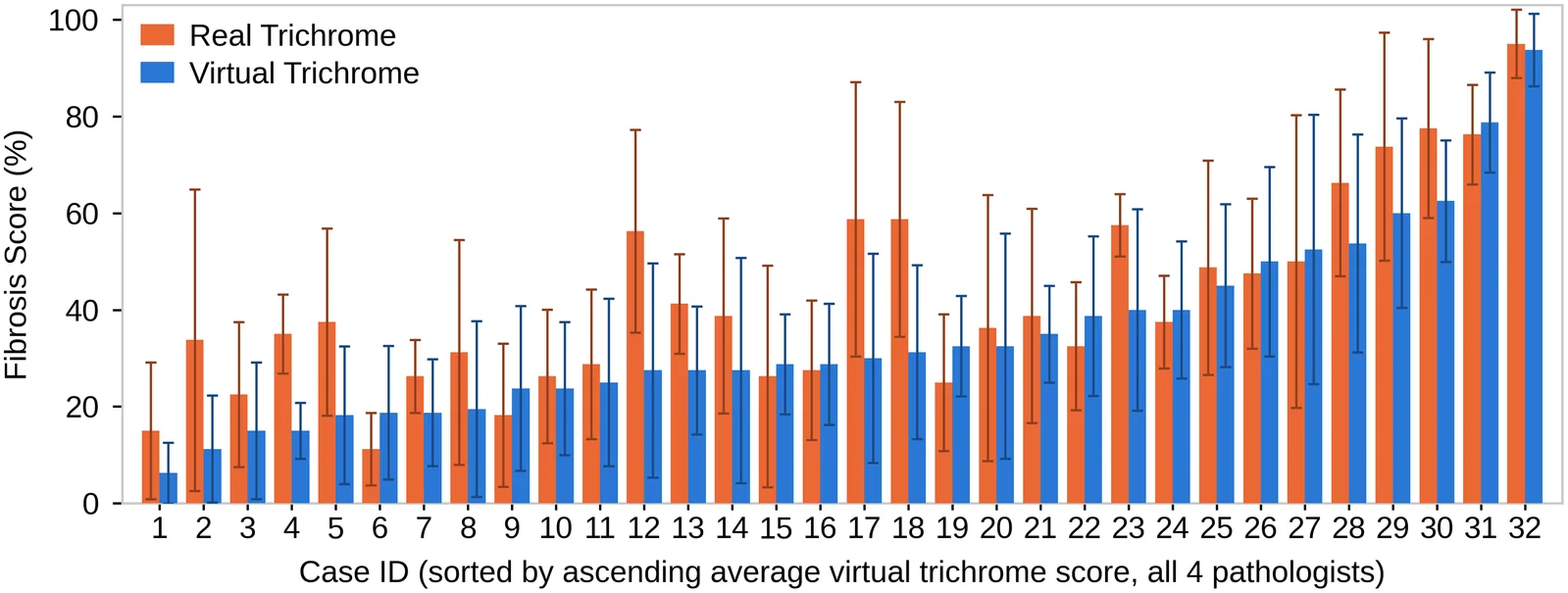
Pathologist fibrosis scores per case in real and virtual trichrome conditions. Bar plots of fibrosis scores for real trichrome (orange) and virtual trichrome (blue) images, averaged across the four pathologists for each of the 32 cases; error bars represent the standard deviation across pathologists. Cases are ordered along the x-axis from left to right based on ascending average virtual trichrome fibrosis score across all four pathologists.

Intraclass correlation coefficients (ICCs) were calculated to assess the agreement of fibrosis estimation for each pathologist when using the real and virtual trichrome WSIs. As seen in Table 2, intra-pathologist ICCs between the two modalities demonstrate moderate to substantial agreement, indicating that the two modalities are comparable for IF estimation.

**Table 2:** Intra-pathologist agreement between real and virtual trichrome.

Pathologist	ICC (95% CI)
A	0.673 (0.427, 0.823)
B	0.767 (0.573, 0.875)
C	0.740 (0.526, 0.860)
D	0.562 (0.272, 0.755)

For each pathologist, the intraclass correlation coefficient (ICC) was calculated between that pathologist’s interstitial fibrosis estimates on the 32 real trichrome whole-slide images and their estimates on the corresponding virtual trichrome whole-slide images. Values therefore reflect how consistently an individual reader scored the same case in the two modalities.

ICCs were also calculated across all four pathologists based on using either real or virtual trichrome WSIs (Table 3).

**Table 3:** Inter-pathologist agreement within each stain modality.

Stain	ICC (95% CI)
Real trichrome	0.477 (0.282, 0.639)
Virtual trichrome	0.536 (0.351, 0.681)

The intraclass correlation coefficient (ICC) was calculated across all four pathologists over the 32 cases, separately for real trichrome and virtual trichrome whole-slide images. Values therefore reflect agreement among readers within a single modality.

A higher inter-pathologist agreement was observed when using virtual trichrome (ICC=0.536) compared to real trichrome (ICC=0.477) WSIs, although this difference was not statistically significant (p=0.376). The same comparison applied to each pathologist’s agreement with the mean estimate of the other three readers (Table 4) showed a higher ICC with virtual trichrome in three of the four pathologists, reaching statistical significance in one (pathologist B, 0.444 versus 0.674, p=0.006).

**Table 4:** Intraclass correlation coefficient (ICC) between individual pathologist fibrosis scores and the mean score of the other three pathologists.

Pathologist	Real trichrome ICC	Virtual trichrome ICC	p-value
A	0.366	0.331	0.795
B	0.444	0.674	0.006
C	0.697	0.711	0.886
D	0.803	0.914	0.111

The reference value excludes the pathologist being evaluated, so it is independent of that pathologist’s own scores. p-values test the difference between the real and virtual trichrome ICCs for that pathologist, and values below 0.05 were considered statistically significant.

Bland–Altman analysis (Fig. 9) showed that real trichrome scores were systematically higher than virtual trichrome scores, by a mean of 7.6 percentage points (paired t test on case-level differences, p=0.0004).

**Fig. 9:**
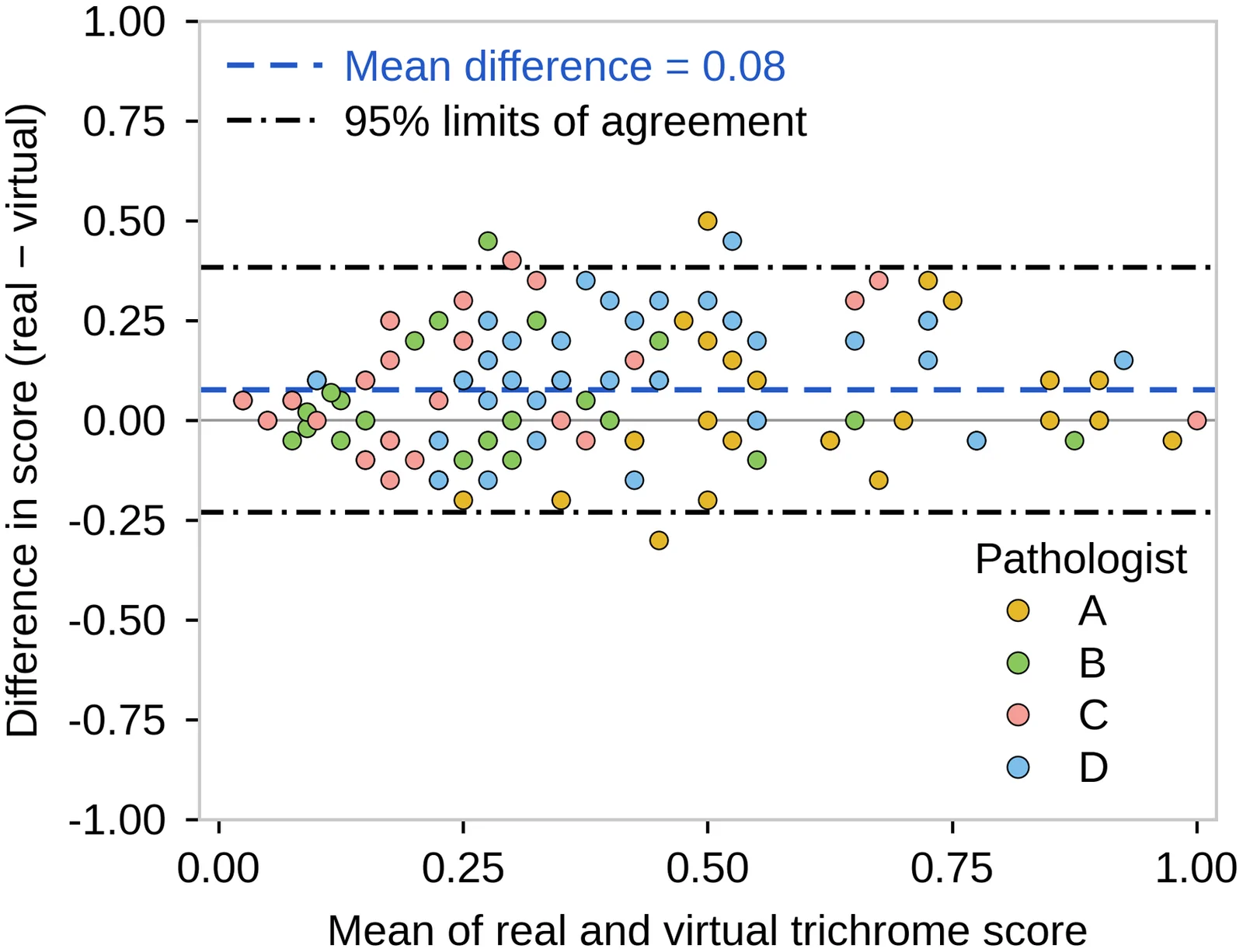
Bland–Altman plot of interstitial fibrosis scores from real and virtual trichrome images. For each paired reading, the difference between scores (real minus virtual) is plotted against the mean of the two scores, with colors indicating each of the four pathologists’ scores. Each point is one pathologist’s paired reading of one case (4 pathologists × 32 cases = 128 pairs). Axes span the full 0 to 1 score scale. The blue dashed line indicates the mean difference (0.08), and the black dashed lines indicate the 95% limits of agreement (−0.23 to +0.38).

The offset was in the same direction for all four pathologists, ranging from 3.2 to 12.7 percentage points, and did not vary with the degree of fibrosis (slope 0.07, p=0.21). The 95% limits of agreement were −0.23 to +0.38 for individual paired readings, narrowing to −0.14 to +0.29 when readings were averaged across the four pathologists within a case (Supplementary Material Fig. S1).

### 3.4 Computational Collagen Quantitation of DUET Images

Collagen images extracted with DUET were used to computationally quantify the degree of IF in WSIs, which was defined as the proportion of collagen-positive pixels within the interstitium relative to the total cortical area. Glomeruli, large arteries, and non-cortical areas were excluded for the computational IF quantitation. Multiclass segmentation enabled exclusion of glomerular and vascular regions prior to fibrosis quantification, ensuring that computational measurements were restricted to the interstitial compartment. These computational quantifications were compared with the average IF estimates of the renal pathologists (Fig. 10).

**Fig. 10:**
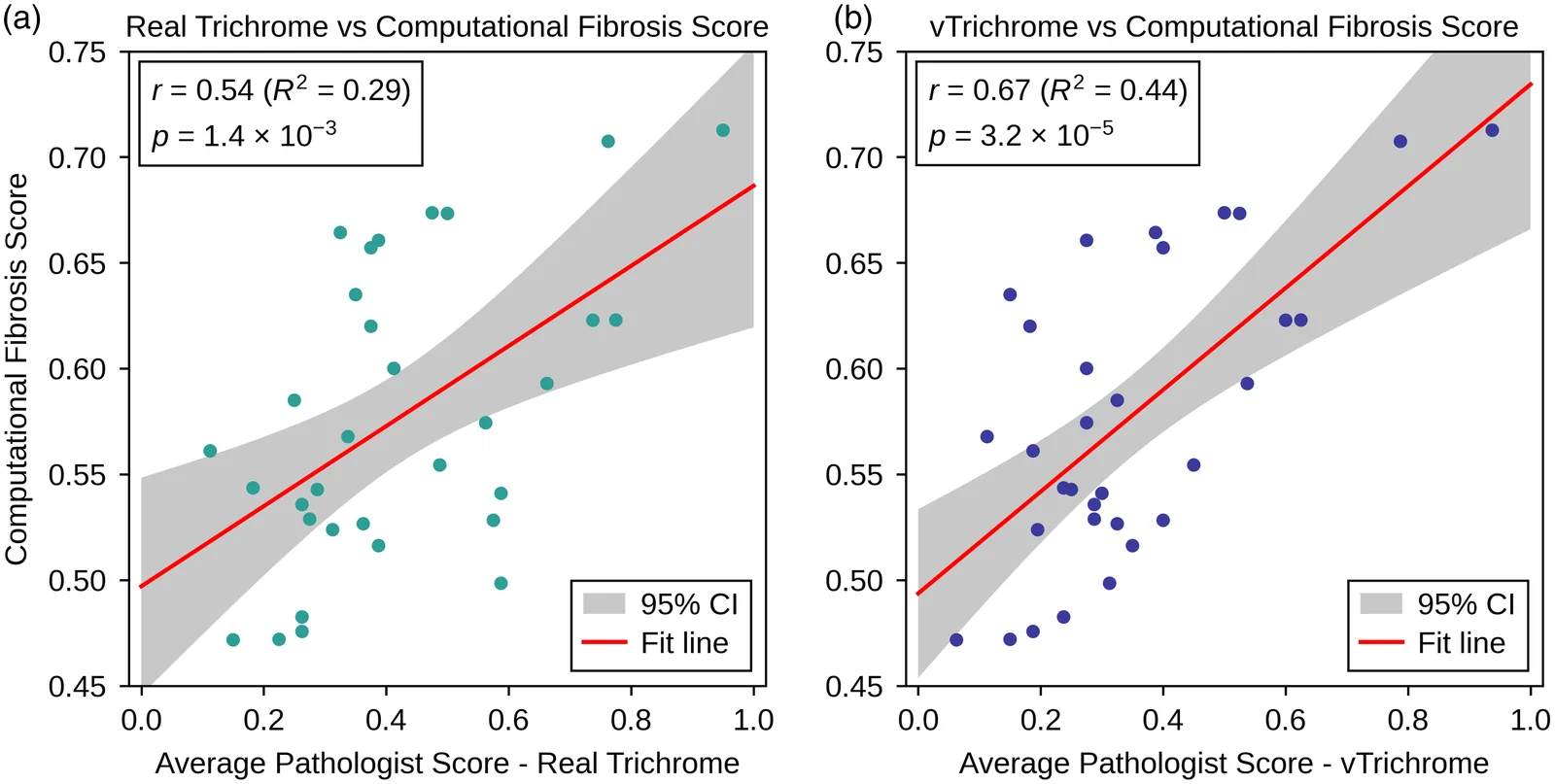
Association between computational fibrosis scores and average pathologist scores for real and virtual trichrome. Scatter plots comparing the computational fibrosis score to the average pathologist score for each case, shown for real trichrome (a) and virtual trichrome (b). Each point represents a single case; the red line represents the least-squares fit, and the gray band indicates the 95% confidence interval. The association with the average pathologist score was stronger for virtual trichrome (r=0.67, $R^{2}=0.44$, $p=3.2\times 10^{-5}$) than for real trichrome (r=0.54, $R^{2}=0.29$, p=0.0014).

The strength of association between pathologists’ estimates and computational IF quantitation, quantified by the Pearson correlation coefficient, was higher for virtual trichrome WSIs (r=0.67, $R^{2}=0.44$) than for real trichrome WSIs (r=0.54, $R^{2}=0.29$), indicating that our computational approach shows a greater alignment with the virtual trichrome modality.

## 4 Discussion

We hypothesized that inter-pathologist agreement with virtual trichrome would be comparable to or better than agreement with conventional trichrome. Agreement among readers was not lower with virtual trichrome than with conventional trichrome (Table 3), and agreement with the mean estimate of the other three readers was higher with virtual trichrome in three of the four pathologists, reaching significance in one (Table 4). Comparable agreement is itself a meaningful result, because virtual trichrome is obtained from the same H&E section used for routine diagnosis, requires no additional stain or tissue, and produces an image that is pixel-registered to the H&E. These advantages hold independently of reader agreement and make this approach worth evaluating in larger cohorts.

This study suggests the potential of DUET imaging to generate clinically useful virtual trichrome stains by extracting collagen-specific fluorescence signals from H&E-stained slides, providing a quantitative framework for automated evaluation of kidney fibrosis and laying the groundwork for future patient outcome prediction. This is achieved through a novel imaging approach that offers a reproducible means of assessing interstitial fibrosis in renal biopsies, closely mimicking traditional trichrome stains while achieving comparable scoring agreement and enabling computational quantification.

Virtual trichrome stains visually recapitulate key histopathological features observed in traditional Masson’s trichrome stains, including collagen deposition patterns associated with IF. The virtual trichrome WSIs demonstrate high reproducibility in qualitative stain consistency, even when input H&E stains exhibit variable quality. Importantly, virtual trichrome WSIs are generated from the same section as the H&E slide rather than an adjacent section, eliminating the risk of missing focal lesions that may not be present in serial sections.

Virtual trichrome scores were on average about 8 percentage points lower than real trichrome scores. One likely reason is that the real trichrome stain also colors interstitial edema and other structures that are not collagen, so it may read higher, while the DUET signal comes from pixels that carry a collagen fluorescence signal. The spread around this average was wide, but it should not be read as error in the virtual trichrome. The two stains were read on different tissue sections, and each case was read only once per stain, so the spread also contains the difference between sections and the normal variation in a single reader’s estimate. Because there is no gold standard for interstitial fibrosis, we cannot say which of the two scores is closer to the truth. The offset is also smaller than the disagreement between pathologists reading the same stain, which averaged 15 to 17 percentage points depending on the stain as the standard deviation across the four readers on a given case (error bars, Fig. 8). What it does mean is that scores from the two stains are not interchangeable on an absolute scale, and whether that carries any practical consequence will need to be settled by comparing both against clinical endpoints such as eGFR trajectory or graft survival.

Given that DUET isolates the collagen signal under fluorescence illumination, we used this signal to generate computational estimates of IF degree. The computational fibrosis score correlated more strongly with virtual trichrome estimates than with real trichrome estimates, likely reflecting their shared source material and differences in tissue sectioning between H&E and serial trichrome slides. The moderate correlation with pathologist estimates in both modalities highlights the computational approach’s potential to align with expert interpretation. The two measures are not on a common scale. The computational score counts all collagen-positive pixels within the interstitial compartment, including normal tubular and glomerular basement membrane, and therefore carries a higher floor than a pathologist’s visual estimate of interstitial fibrosis. This is why the comparison is reported as strength of association rather than agreement. Although we excluded non-tubulointerstitial structures such as glomeruli and larger arteries using a multiclass segmentation model, the primary role of this pipeline was not for precise segmentation, but rather coarse exclusion of nontubular areas.

Virtual trichrome images have previously been generated from brightfield H&E using generative adversarial networks in supervised paired^16^ and unsupervised unpaired^17^ settings. de Haan et al. used collagen-informative autofluorescence to build registered training targets,^16^ but that signal is available only during training; at inference the network receives H&E alone, so collagen is predicted from the H&E appearance rather than measured in the section at hand, and reported failures include trichrome positivity generated where no collagen is present.^17^ The DUET pipeline also uses a learned model, and segmentation errors produce false-positive and false-negative regions. The distinction is that the collagen-specific fluorescence signal is acquired for every case and supplied as input, so segmentation is conditioned on a signal physically related to collagen in that section. Errors therefore appear as mislabeled regions of real tissue rather than as histological structures that are not present in the section, which generative models have a real risk of producing.

Interstitial fibrosis is a common final pathway of chronic injury across renal diseases; the matrix deposited consists largely of the same collagen types regardless of etiology, and the DUET signal derives from the intrinsic fluorescence of that matrix rather than from any transplant-specific feature. We would therefore not expect the method to behave differently in native kidney biopsies, but this was not evaluated here and is a target for future work.

The system used in this study is a laboratory-built microscope, but the capability does not depend on custom hardware. Fluorescence-enabled whole-slide scanners are already commercially available, and the additions required to acquire DUET images on a conventional brightfield scanner are modest: an epifluorescence illumination path, a 405 nm LED, and the corresponding excitation and emission filters. Hardware cost for our prototype was approximately $25,000, and because the modifications are simple, a commercially integrated implementation would be expected to remain in the price range of current whole-slide scanners. Acquisition is slower than brightfield scanning alone, since two images are collected at each field of view rather than one, requiring approximately 5 to 15 min per slide for a typical core biopsy. We consider this trade-off favorable, as the additional acquisition time yields a collagen-specific image and a pixel-registered virtual trichrome from the same section, with no additional sections, stains, or reagents.

A limitation of our study is the lack of a widely accepted “gold standard” for IF. Although the pathologists’ visual estimation is often used, this type of subjective evaluation is plagued by both inter- and intra-pathologist variability. The four pathologists in this study are from three different academic institutions and trained at four different training programs, which likely contributed to some degree of inter-pathologist variability. The mean of the four estimates was therefore used as a reference standard where a single pathologist score was not appropriate, and in Table 4 the reference for each pathologist excludes that pathologist’s own scores so that it is independent of them. We also used second harmonic generation, an accepted method for collagen imaging, to establish a reference standard for the collagen signal extracted from DUET.

Collagen ground truth was derived from spectral phasor cluster assignments that were reviewed by the study team and confirmed under renal pathologist guidance. Noncortical regions were excluded by manual pathologist annotation, and glomeruli and large arteries were excluded by a segmentation model trained on study-team annotations. These steps carry a degree of operator subjectivity, and the segmentation metrics reported in Table 1 therefore reflect agreement between the model and this reference rather than against an absolute ground truth. No absolute standard exists for interstitial collagen, as no single stain or imaging modality captures every collagen type relevant to interstitial fibrosis.

The cohort was assembled to span the full range of interstitial fibrosis, with enrichment for cases of advanced fibrosis, and therefore does not reflect the case mix of routine transplant practice. This does not affect the comparison between modalities, since every case was scored by the same four pathologists in both conditions and selection was performed on the original slides before any virtual trichrome image was generated. It does mean that the absolute agreement values reported here should not be read as expected performance on a consecutive clinical series. Extrapolation to other centers will require validation in a multi-institution cohort of clinical biopsies.

Each pathologist scored each case once per modality, so intra-observer reproducibility was not measured here. A single read reflects routine practice, in which the fibrosis estimate that reaches the clinician comes from one pathologist reading the slide once. Variability in visual fibrosis scoring has been characterized in dedicated reproducibility studies.^10^^,^^11^ Quantifying it would require a test-retest arm, in which a subset of cases is re-read by the same pathologist after a washout interval.

It is also important to emphasize that both visual and computational assessments in this study primarily reflect interstitial expansion rather than biochemically isolated fibrosis. Interstitial expansion may result from fibrosis, edema, inflammation, or basement membrane thickening, which cannot be fully disentangled by conventional or virtual trichrome staining alone. This limitation is shared by traditional trichrome-based assessment and reflects a broader challenge in renal pathology rather than a constraint unique to DUET. In addition, evaluation of fibrosis maturity and matrix composition, which may depend on subtle chromatic differences in conventional trichrome stains, was not addressed in this study. Importantly, the virtual trichrome signal in DUET is not generated solely by morphologic delineation of interstitial spaces but is derived from fluorescence-based collagen signal that is pixel-registered to the underlying H&E architecture. Although pseudo-coloring is applied for visualization, the spatial distribution and intensity of the virtual stain are determined by measured optical signal rather than arbitrary color placement. Future studies incorporating multi-channel fluorescence or spectral metrics may further enable discrimination of edema, early fibrosis, and mature matrix composition.

## 5 Conclusion

Taken together, these results suggest that DUET imaging, particularly when paired with computational analysis, holds promise as a reproducible and scalable adjunct to traditional histology. Future prospective studies should explore its impact on clinical outcomes, as well as its integration with digital workflows for remote, frozen section, or high-throughput pathology settings. Beyond replication of conventional staining, DUET enables the generation of collagen images that are precisely pixel-registered to diagnostically informative brightfield images. This spatial alignment preserves morphologic context and may enhance computational model performance, facilitating accelerated discovery of collagen-based biomarkers and supporting large-scale predictive models for chronic kidney disease progression and transplant outcomes.

## Supplementary Material

10.1117/1.JMI.13.6.067501.s01

## Data Availability

The DUET imaging system used in this study is a custom-built dual-mode emission and transmission microscope. Hardware configuration and acquisition parameters are described in the Methods section (Sec. 2). The whole-slide imaging datasets generated in this study are not publicly available due to institutional data governance and patient privacy restrictions. De-identified data may be shared with qualified investigators subject to approval by the UC Davis Institutional Review Board and execution of any required data use agreements. Requests should be directed to the corresponding author and will be reviewed in accordance with institutional policies. The computational pipeline, including deep learning model architecture and analysis workflows for collagen segmentation and virtual trichrome generation, is based on custom-developed code for the DUET system. Code access may be provided to researchers for non-commercial research use under appropriate agreements; details are available from the corresponding author.
