# Supplementary material for: Kidney fibrosis evaluation using dual-mode emission and transmission microscopy
Source: J Med Imaging (Bellingham). 2026 Nov 5;13(6):067501. doi: 10.1117/1.JMI.13.6.067501 (PMC13735734; doi:10.1117/1.JMI.13.6.067501)
Supplement: Supplementary file 1 [file JMI_013_067501_SD001.pdf]

Supplementary Material

Table S1 | Machine learning steps and their training datasets.

	**Collagen segmentation**	**Glomeruli and artery segmentation**
**Purpose**	Extract collagen-positive pixels for the virtual trichrome image and computational fibrosis score	Exclude glomeruli and large arteries from the computational fibrosis score only
**Model**	UNet++	U-Net
**Inputs**	Green channel of pixel-registered brightfield and fluorescence images, 512 × 512 patches	Green channel of pixel-registered brightfield and fluorescence images, 4096 × 4096 tiles downsampled to 1024 × 1024
**Ground truth**	Collagen masks from the supervised spectral phasor approach, verified against SHG imaging and pathologist guidance (K.-Y.J.)	Manual annotation of glomeruli and arteries in FIJI/ImageJ
**Dataset**	700 patches from 3 cases spanning a range of fibrosis	363 patches from 4 cases
**Train / test split**	80% training, 20% held out for evaluation (Table 1)	252 training, 111 validation (stratified 70/30)

Summary of the two machine learning models used in this study, the data on which each was trained, and the source of the corresponding ground truth labels.

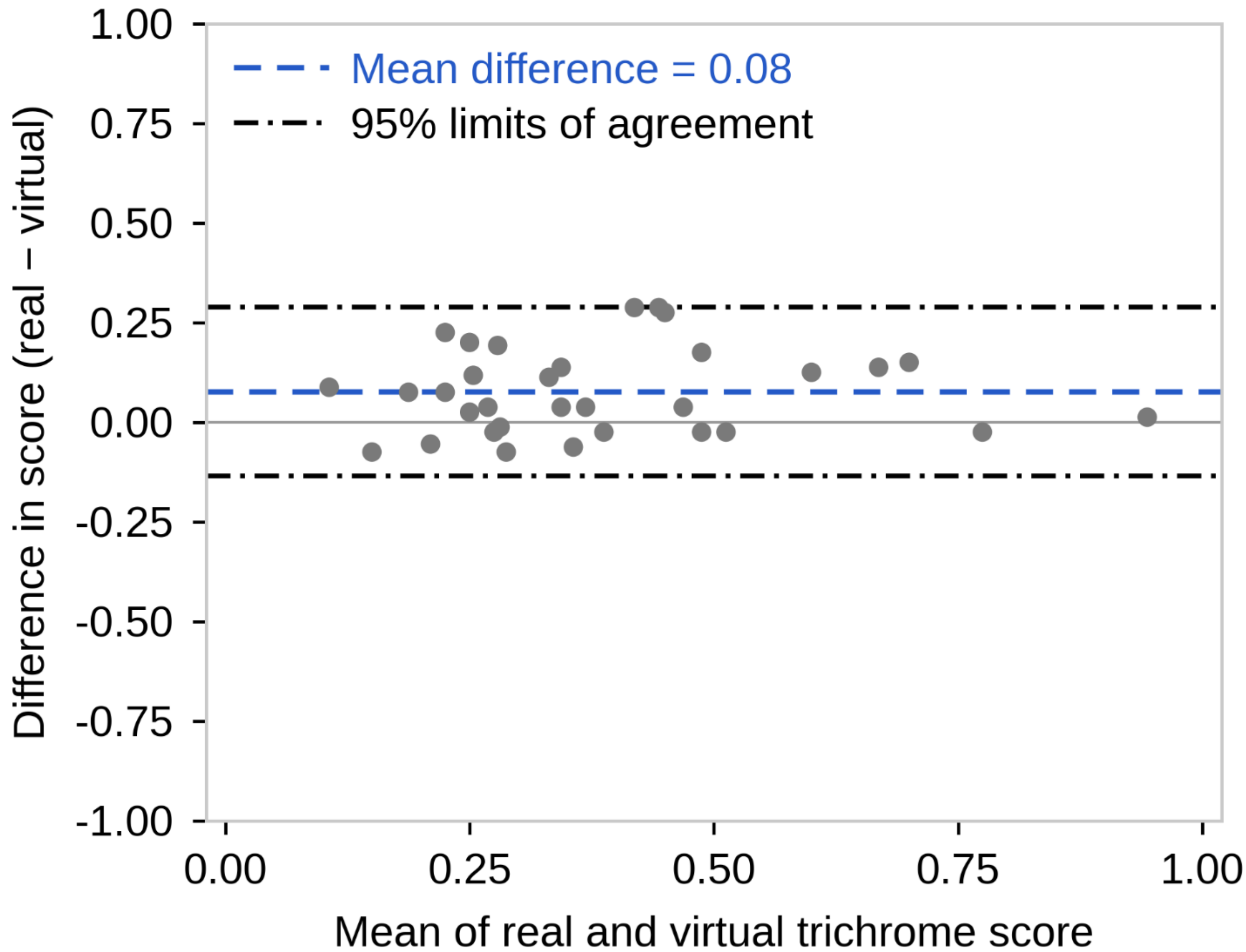

Figure S1. Case-level Bland-Altman plot of interstitial fibrosis scores from real and virtual trichrome images. Each point is one case (n = 32), with the difference between the real and virtual trichrome score (real minus virtual) averaged across the four pathologists and plotted against the mean of the two averaged scores. Axes span the full 0-1 score scale. The blue dashed line indicates the mean difference (0.08) and the black dashed lines the 95% limits of agreement (−0.14 to +0.29).
